# Probing calcium solvation by XAS, MD and DFT calculations†

**DOI:** 10.1039/d0ra05905f

**Published:** 2020-07-21

**Authors:** Feipeng Yang, Yi-Sheng Liu, Xuefei Feng, Kun Qian, Li Cheng Kao, Yang Ha, Nathan T. Hahn, Trevor J. Seguin, Mesfin Tsige, Wanli Yang, Kevin R. Zavadil, Kristin A. Persson, Jinghua Guo

**Affiliations:** Joint Center for Energy Storage Research Lemont IL 60439 USA; Advanced Light Source, Lawrence Berkeley National Laboratory Berkeley CA 94720 USA; Department of Polymer Science, The University of Akron Akron OH 44325 USA; Material, Physical and Chemical Sciences Center, Sandia National Laboratories Albuquerque NM 87185 USA; Energy Technologies Area, Lawrence Berkeley National Laboratory Berkeley CA 94720 USA; Department of Materials Science and Engineering, University of California, Berkeley CA 94720 USA; Department of Chemistry and Biochemistry, University of California Santa Cruz CA 95064 USA jguo@lbl.gov

## Abstract

The solvation shell structures of Ca^2+^ in aqueous and organic solutions probed by calcium L-edge soft X-ray absorption spectroscopy (XAS) and DFT/MD simulations show the coordination number of Ca^2+^ to be negatively correlated with the electrolyte concentration and the steric hindrance of the solvent molecule. In this work, the calcium L-edge soft XAS demonstrates its sensitivity to the surrounding chemical environment. Additionally, the total electron yield (TEY) mode is surface sensitive because the electron penetration depth is limited to a few nanometers. Thus this study shows its implications for future battery studies, especially for probing the electrolyte/electrode interface for electrochemical reactions under *in situ*/operando conditions.

## Abstract 2

The solvation shell structures of Ca^2+^ in solutions are probed by calcium L-edge soft X-ray XAS and DFT/MD simulations.

## Graphical abstract

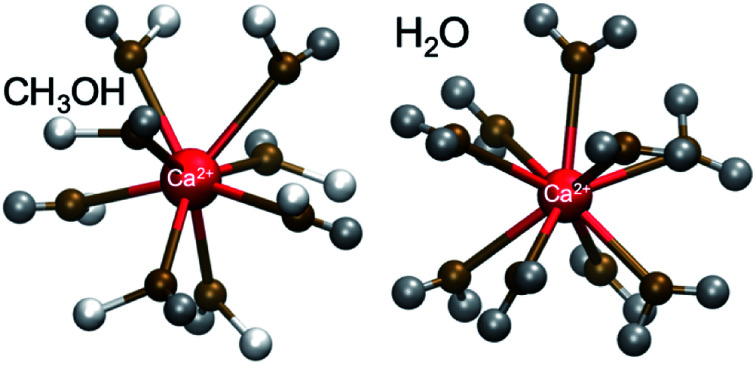

## Introduction

Calcium, as the fifth abundant element in the earth crust,^1^ not only plays a significant role in many geologic and biogenic processes, but also is a promising candidate for “beyond-lithium-ion” batteries with the potential to revolutionize energy storage. Geologically, calcium is a major component of ground and saline waters and many other geologic formations.^2^ Biochemically, the calcium ion is the most abundant cation in the human body, and plays an essential part in the process of nerve action and muscle.^3^ Calcium carbonate is a major mineral formed in various biomineralization processes, such as the grinding tip of the sea urchin tooth,^4^ sea urchin larval spicule,^5^ and coral skeletons.^6^ With five different crystalline polymorphs, anhydrous phases and hydrated phases, the mechanism of such phase transitions has been investigated systematically using X-ray absorption spectroscopy (XAS) and photoelectron emission spectromicroscope.^7–9^ From the perspective of energy science, calcium oxide works as a catalyst in the production of biodiesel, part of which process is due to the dissolution of the activated CaO in methanol that creates homogeneous leached active species.^10^ As a promising candidate in multivalent battery technologies, it offers the promise of more than two-fold increase in the volumetric capacity compared to the monovalent lithium-ion batteries.^1^ Being nontoxic, safe and economic, and with a comparable cell voltage and energy density, calcium battery serves as an attractive candidate for “beyond-lithium-ion” battery technologies.^11,12^

Common to the applications mentioned above is the fundamental question of the calcium environment, *e.g.* solvation in both aqueous and non-aqueous solutions. Ions in solution are usually coordinated primarily by solvent molecules, interacting *via* electrostatic forces and/or hydrogen bonds. However, in electrochemical systems, facile solvation and desolvation at an electrified interface is necessary for continuous operations. Here we present a molecular-scale study, at relevant (nanometer) distances from an interface, which is of crucial importance for understanding such solvation phenomena. An understanding of the ion solvation and its variation at the atomic level will allow adjusting the ion solubility, reactivity and stability independently, leading to breakthroughs in battery and electrochemical science.^13,14^

As an important topic in understanding battery materials, the investigation of calcium solvation has started early in the 1980s. Probst and coworkers used molecular dynamics (MD) calculations and X-ray scattering methods to study 1.1 M aqueous CaCl_2_ aqueous solution, and found a total of 33 water molecules are engaged in either the first or second coordination sphere, while there is a large overlap between the coordination shells of different ions.^15^ Later in 2003, calcium K-edge absorption spectroscopy was used by Fulton *et al.* to probe the effects of concentration variations on the first-shell structure of Ca^2+^ in aqueous solution.^16^ A mean coordination number of 7.2 ± 1.2H_2_O molecules and an average Ca–O distance of 2.437 ± 0.010 Å were derived from 6 M CaCl_2_ aqueous solutions. An accurate description of calcium solvation in concentrated aqueous solutions was developed in 2014 using a novel force field model in MD calculations by Kohagen and coworkers.^17^ A few years later, a combination of *ab initio* MD simulations and neutron scattering experiments were employed in the characterization of ion hydration and pairing in aqueous calcium chloride and formate/acetate solutions by Martinet *et al.*^18^ In a separate study by Guo *et al.*, the solvent effect for the solvation structure of Ca^2+^ in polar molecular liquids were studied systematically using calcium K-edge X-ray absorption spectroscopy (XAS).^19^ In that work the liquid samples are sealed in mylar films and the bulk properties are being probed, due to the penetration depth of fluorescence is on the order of hundreds of nanometers. The calcium L-edge soft XAS was reported to study different types of solid calcium compounds, such as CaCl_2_·2H_2_O, CaF_2_, CaCO_3_, *etc.*,^20^ but with very limited reporting on using calcium L-edge soft XAS to study calcium solvation environment in solutions.

Synchrotron based XAS measures the X-ray absorption coefficient as a function of the incident X-ray photon energy in a range below and above the absorption edge of a particular element.^21^ As a highly element specific technique with sensitivity to the local chemical environment and structural order of the element of interest, it is an impressive tool in probing the oxidation state, bond length and coordination.^22–25^ As one of the two categories in XAS, studies that investigate the region from the absorption edge to tens of eV above the edge are designated as near edge X-ray absorption fine structure (NEXAFS). Detailed electronic information on the unoccupied states near the Fermi level, which is strongly affected by the solvation structure, can be derived from XAS.

In this article, both L-edge soft XAS and simulation methods including MD and density functional theory (DFT) calculations are used to scrutinize the calcium solvation in aqueous and organic solutions. The L-edge soft XAS is more sensitive to the local chemical environment of calcium compared to that from hard X-ray K-edge measurement since it probes the excitation of 2s or 2p electrons while the K-edge measures the 1s electrons. In contrast to our earlier work probing the bulk properties,^19^ the L-edge XAS measured using our specially designed *in situ*/operando cell enables us to apply electrochemical potentials while XAS intensities being collected, making *in situ*/operando observation of the electrode/electrolyte interface possible, which dictates the battery performance. It benefits from the surface sensitive nature of the total electron yield (TEY) mode in the soft X-ray L-edge XAS of calcium, which limits the probing depth within 1–2 nm. This manuscript is not only reporting the results of CaCl_2_ solvation but the method itself will be of significance in battery and catalysis areas. The L-edge soft XAS is unique, and will help reveal the solvation process at the electrode/electrolyte interface in nanoscale, aiding in the development of “beyond-lithium-ion” calcium batteries.

## Experimental section

The calcium L_3,2_-edge XAS spectra were measured at Advanced Light Source (ALS) beamlines 7.3.1 and 8.0.1 in Lawrence Berkeley National Laboratory. The storage ring condition is 1.9 GeV and 500 mA current in a multi-bunch operation mode. The XAS spectra were collected in total electron yield (TEY) and total fluorescence yield (TFY) modes and calibrated using solid CaCl_2_. An *in situ*/operando flow liquid cell at beamline 8.0.1.4 (wetRIXS endstation) was used in the data collection. The TEY mode XAS limits the penetration depth to 1–2 nm from the electrode/electrolyte interface, thus it is not affected by the substantial interference from the bulk liquid. Combined with the flow liquid cell, it enables the probing of transient state species at the electrode/electrolyte interface under *in situ*/operando conditions.

Molecular dynamics (MD) simulation was used to study the hydration of calcium ions in methanol and aqueous solutions. The force field parameters used in the simulation were derived by fitting the parameters of suitable analytical functions on the *ab initio* energy points obtained from Hartree–Fock calculations for CaCl_2_ in H_2_O^26^ and methanol.^27^ All non-Coulomb interactions involving ions are expressed through:*V*_ij_(*r*) = *A*_ij_/*r*_ij_^*n*_ij_^ + *B*_ij_ exp(−*C*_ij_*r*_ij_)where *r*_ij_ denotes the distance between two ions or between an ion and a solvent atom. The parameters *n*_ij_, *A*_ij_, *B*_ij_, and *C*_ij_ were employed to describe non-Coulomb interactions, which could be found in ref. 26 and 27. Regarding the solvents, we used OPLS rigid three-site methanol model^28,29^ and SPC/E water model,^30^ which were used by Migliorati *et al.*^31^ in simulation work of the hydration of Zn^2+^ in aqueous and methanol solutions that resulted in good agreement with XAS experimental data. To simulate different concentrations of calcium ions in a solution: 10, 20, 30, and 60 CaCl_2_ molecules were added in a simulation box containing 1112 water molecules to represent 0.5, 1.0, 1.5, and 3.0 mol L^−1^ CaCl_2_ aqueous solutions, respectively. Similarly, 10 and 20 CaCl_2_ molecules were added in a simulation box containing 497 methanol molecules to represent 0.5 and 1.0 mol L^−1^ CaCl_2_ methanol solutions, respectively. Periodic boundary conditions in all three directions have been applied during all the simulations.

The radial distribution functions (RDF), *g*_AB_(*r*), which gives the probability of finding atom B at a distance *r* from atom A, was calculated using the expression:
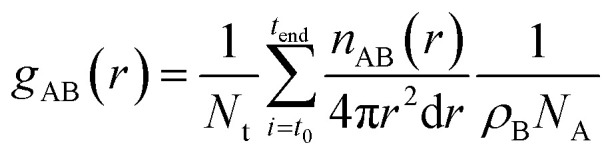
where *n*_AB_(*r*) is the number of B atoms at a distance between *r* and *r* + d*r* away from atom A, *ρ*_B_ is the density of B atoms in the simulation box, *N*_A_ is the total number of A atoms in the simulation box, and *N*_t_ is the total number of frames in 1 ns. Thus, *ρ*_B_ × *g*_AB_(*r*) represents the absolute density of B at a distance between *r* and d*r* and is expressed as:
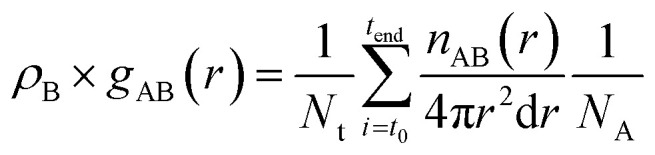


DFT calculations were carried using ORCA packaging on the supercomputing cluster Lawrencium at Lawrence Berkeley National Laboratory. The initial guessed structures were based on the pre-optimized MD simulation results, using Ca^2+^ only surrounded by H_2_O to model very diluted case, and including Cl-in the solvation environment of Ca^2+^ to model relative concentrated case. B3LYP functional and TZVP basis set were used under PCM water, and the ROCIS calculations were applied to simulate the Ca L-edge spectra.

## Results and discussion

**Fig. 1:**
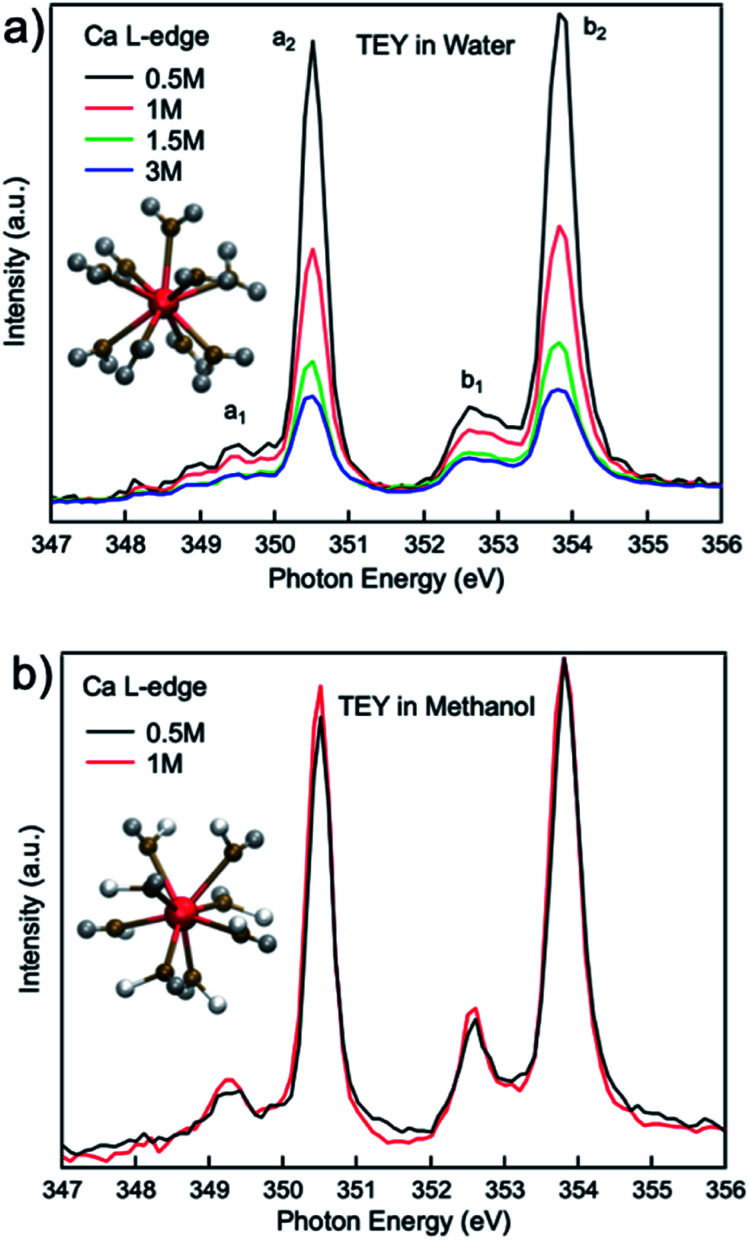
Calcium L_3,2_-edge TEY mode XAS spectra for (a) 0.5 M, 1.0 M, 1.5 M, and 3 M CaCl_2_·2H_2_O aqueous solutions; (b) 0.5 M, and 1 M CaCl_2_·2H_2_O methanol solutions.

Fig. 1(a) compares the XAS from 0.5 M, 1 M, 1.5 M, and 3 M CaCl_2_·2H_2_O aqueous solutions from TEY mode. It is shown that the calcium L_3,2_-edge XAS spectrum is composed of two main spin–orbit related peaks (a_2_ and b_2_) corresponding to L_3_ and L_2_-edge, while two smaller peaks (a_1_ and b_1_) precede the L_3_ and L_2_-edge main peaks.^20,32^ The two main peaks are the 3d electrons in t_2g_ symmetry:^33^ a_2_ and b_2_ are associated with the 2p_3/2_^−1^t_2g_ and the 2p_1/2_^−1^t_2g_ states, respectively. The smaller peaks are 3d electrons in e_g_ symmetry: a_1_ and b_1_ are associated with 2p_3/2_^−1^e_g_ and 2p_1/2_^−1^e_g_ states, respectively. The origin of the multipeak pattern is crystal field splitting, resulting from the magnitude and symmetry of the crystal field of calcium in the first coordination sphere.^34^ It is also revealed that the intensity of the peaks is inversely correlated with the CaCl_2_·2H_2_O concentration. In contrast, for 0.5 M and 1 M CaCl_2_·2H_2_O in methanol, the overall spectra are similar, indicating that an increased ratio of the Ca^2+^ cations to methanol molecules does not affect the Ca^2+^ cation chemical coordination.

**Fig. 2:**
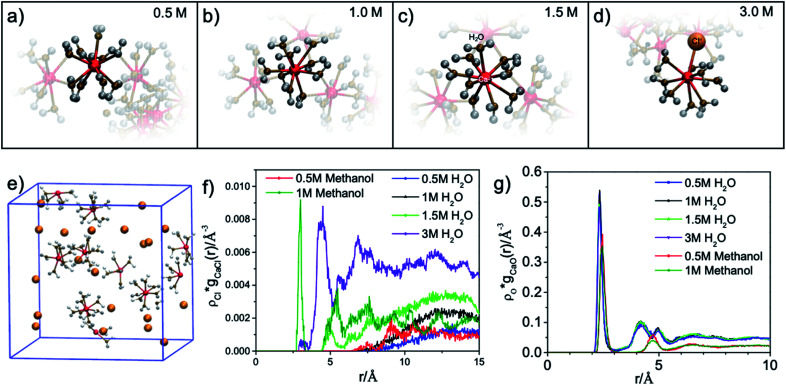
Rendered single Ca^2+^ and its first solvation shell from MD simulations for (a) 0.5 M, (b) 1.0 M, (c) 1.5 M and (d) 3.0 M aqueous solutions. (e) Molecular dynamics (MD) simulations for the first solvation shell around Ca^2+^ in 0.5 M CaCl_2_·2H_2_O methanol solution. (f and g) Absolute density distribution of chloride ion (Cl^−^) and oxygen atom (O) as a function of the distance from Ca^2+^, respectively.

The results of the molecular dynamics (MD) simulations indicate that the average distance between Ca^2+^ and oxygen atoms in its first solvation shell is 2.32 ± 0.01 Å in aqueous solutions and 2.44 ± 0.02 Å in methanol solutions (see Fig. 2(g)). It was also observed that in the second solvation shell (Fig. S1–S3†), all the oxygen atoms in the methanol solutions are, on average, found at the same distance of 4.74 ± 0.02 Å from the Ca^2+^ while the oxygen atoms in aqueous solutions are either at 4.25 ± 0.01 Å or 4.97 ± 0.02 Å from the Ca^2+^. For the 0.5 M methanol solution, 0.5 M and 1 M aqueous solutions, no Cl^−^ is observed within 5 Å from the Ca^2+^, while for the 1 M methanol solution, 1.5 M and 3 M aqueous solutions, Cl^−^ anions are observed at distances less than 5 Å from Ca^2+^ and unevenly distributed as a function of distance (see Fig. 2(f)). In the diluted (0.5 M) CaCl_2_·2H_2_O methanol solution, Ca^2+^ cations are separated from each other with a distance beyond the average distance from Ca^2+^ cation to the oxygen atoms in its first solvation shell. However, even in 0.5 M CaCl_2_·2H_2_O dilute aqueous solution, dimers and ionic clusters form by sharing the H_2_O molecules in their first solvation shell. Such dimers and ionic clusters are also observed in the more concentrated CaCl_2_·2H_2_O aqueous solutions (1.0 M, 1.5 M, and 3 M). In aqueous solutions, with an increase of Ca^2+^ cation concentration, the solvent coordination number is the same while the nearby Ca^2+^ cation and Cl^−^ anion influence is increasing, as shown in Fig. 2(a–d). For 3 M aqueous solution in Fig. 2(d), one Cl^−^ anion appears in the first solvation shell, replacing one H_2_O molecule. For methanol solutions of different concentrations, the solvation structures are similar, an example is shown in Fig. 1(b). The equilibrium number of solvent molecules surrounding the Ca^2+^ cation, are 8 and 9 in its first solvation shell in methanol and aqueous solutions, respectively. Such Ca complexes with coordination numbers of 8 or 9 have not been reported in the crystallographic database. The MD simulation also found that in aqueous solutions, each H_2_O molecule in the first solvation shell exhibits three neighbouring H_2_O in the second shell, connected by hydrogen-bond and forming tetrahedral networks. The higher the CaCl_2_·2H_2_O concentration, the smaller the hydrogen-bond network scale with respect to each Ca^2+^. Since such influence is beyond the first solvation shell, the XAS spectrum intensity is sensitive to the CaCl_2_·2H_2_O concentration. The density distribution of oxygen atoms in Fig. 2(g) shows tiny variations because the relative value of change with respect to the total value is too small thus hard to be observed. The reason is that there are many oxygen atoms in the aqueous solution while those affected by the hydrogen bond constituent only a fraction of the oxygen atoms in H_2_O molecules. In contrast, the total number of chloride ions is small, thus those affected by the hydrogen bonding are easy to be observed, as it is shown in Fig. 2(f). In contrast, in the methanol solution each CH_3_OH molecule in the first solvation shell exhibits only one neighbouring CH_3_OH molecule in the second shell, with weaker interaction because of weaker polarity and steric hinderance. As a result, the XAS spectra are comparable from CaCl_2_·2H_2_O methanol solution of different concentrations.

**Fig. 3:**
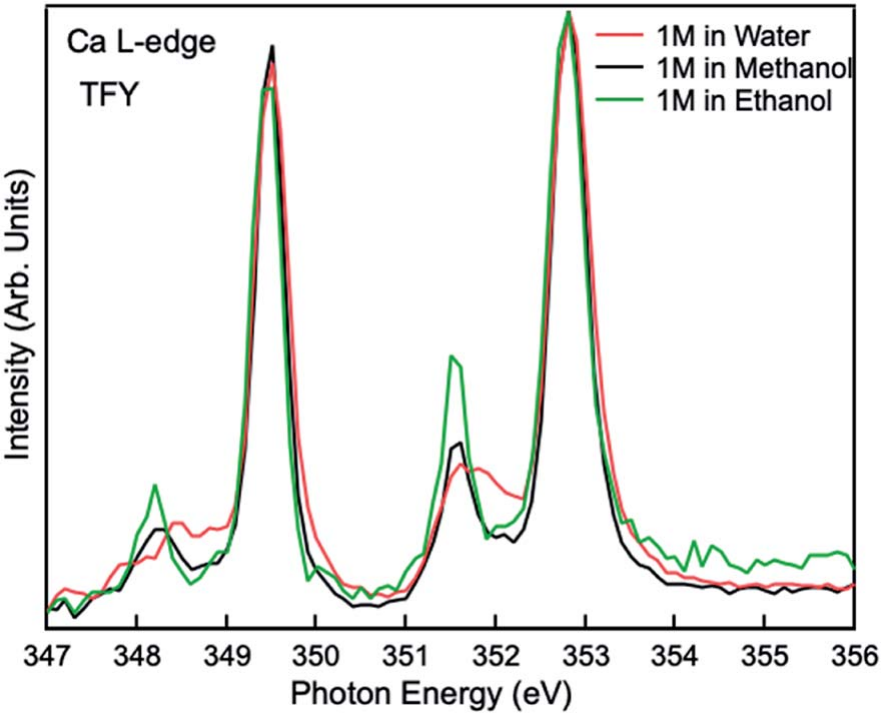
Calcium L_3,2_-edge TFY mode XAS spectra for 1 M CaCl_2_·2H_2_O in H_2_O, methanol, and ethanol.

Comparisons are also made between 1 M Ca^2+^ in H_2_O, methanol and ethanol solutions collected using TFY mode. The two main peaks (∼349 and 353 eV) have identical features in terms of intensity and full width half maximum (FWHM), while the two smaller peaks (a_1_ and b_1_) precede the L_3_ and L_2_-edge main peaks differ from each other. The intensity for both pre-edge peaks become larger and the FWHM become smaller following the sequence of H_2_O, methanol, and ethanol solution, as it is shown in Fig. 3. It indicates that for solvent with weaker polarity and larger steric hinderance, the intensity of the pre L_3,2_-edge peak will become larger and the FWHM of this pre-edge peak will become smaller, due to a decrease in the number of oxygen atoms surrounding the Ca^2+^ cations and its corresponding influence on the 3d electrons in e_g_ symmetry.

**Fig. 4:**
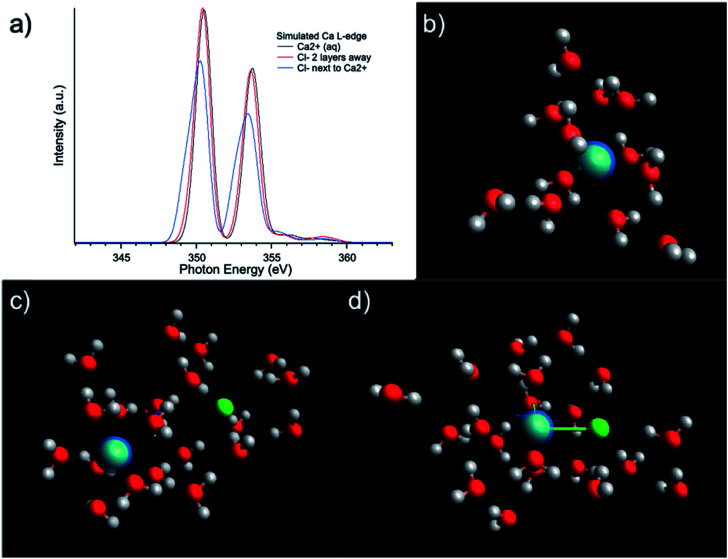
(a) L_3,2_-edge spectra calculated using ROCIS calculations based on models of (b) Ca^2+^ + 12H_2_O representing dilute solution; (c) Ca^2+^ + Cl^−^ + 20H_2_O with two molecular layer separation between Cl^−^ and Ca^2+^; (d) Ca^2+^ + Cl^−^ + 20H_2_O with Cl^−^ in the first solvation shell of Ca^2+^ representing a high concentration solution.

The influence of solute concentration on the calcium L_3,2_-edge spectrum was also confirmed by DFT calculations. Using initial guessed structures based on the pre-optimized MD simulation results, three cases were compared as shown in Fig. 4. The Ca^2+^ only surrounded by H_2_O in Fig. 4(b) represents very diluted case, and cases in Fig. 4(c and d) including Cl^−^ in the solvation environment of Ca^2+^ to model relative concentrated case. Fig. 4(b–d) represent Ca^2+^ aqueous solutions of increasing concentrations. The L_3,2_-edge spectra calculated from Fig. 4(b–d) are shown in Fig. 4(a). They are consistent with the experimental data shown in Fig. 1(a) that with a higher Ca^2+^ concentration, the intensity of the Ca^2+^ L_3,2_-edge spectrum will become lower.

**Fig. 5:**
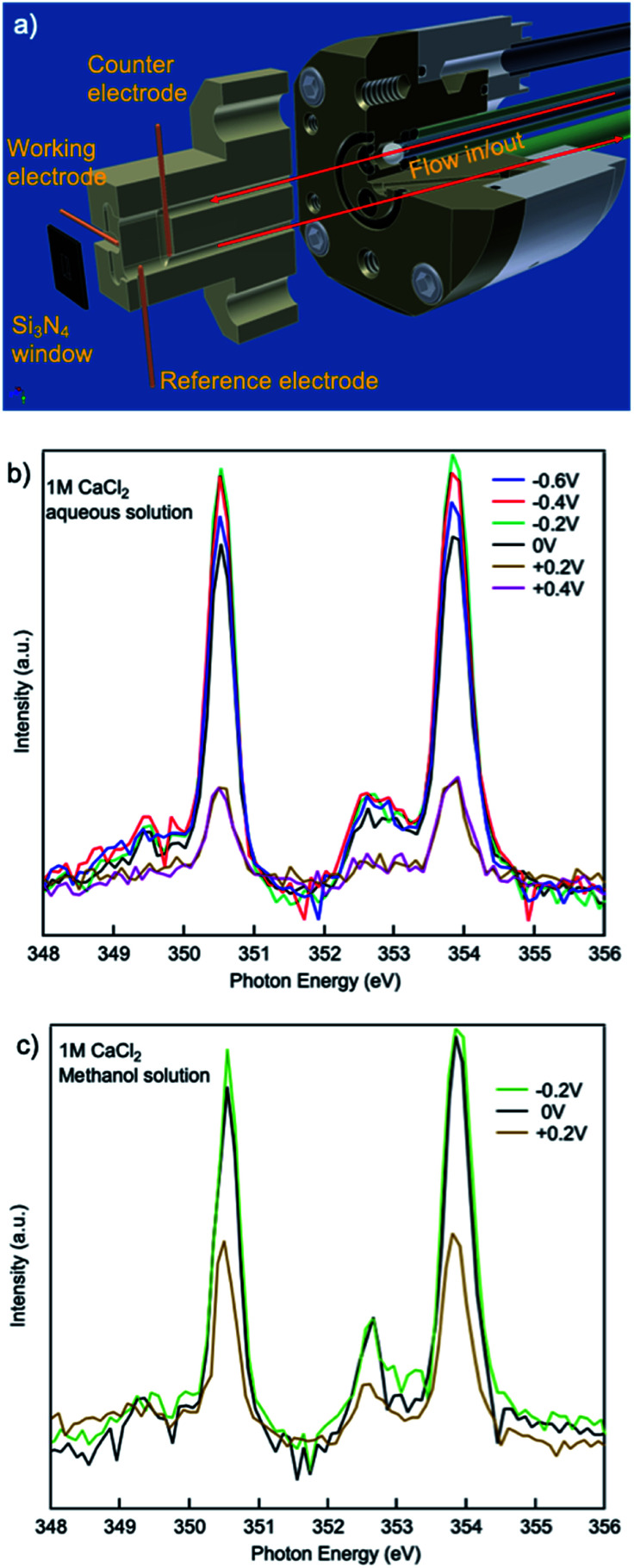
(a) Schematic of *in situ*/operando flow cell at beamline 8.0.1.4. XAS spectra from 1 M CaCl_2_·2H_2_O (b) aqueous and (c) methanol solutions with different potentials applied on the working electrode.

The effect of electrochemical potential applied was evaluated using an *in situ*/operando flow cell at beamline 8.0.1.4 (wetRIXS endstation), a schematic of the drawing is in Fig. 5(a). Potentials ranging from −0.6 V to +0.4 V were applied on the working electrode while TEY was being collected. For a CaCl_2_·2H_2_O aqueous solution with no potential or negative potentials applied, the spectra are similar in the a_1_, b_1_ small peaks preceding the L_3,2_-edge. Interestingly, at the L_3,2_-edges, when no potential is applied the spin–orbit related a_2_, b_2_ peaks exhibit the lowest intensity, while when negative potentials are applied, a_2_, b_2_ peak intensities are always larger, and the smaller the absolute potential is applied, the larger the intensity. This change in intensity originates from the electrostatic interactions between the working electrode and the Ca^2+^ cations in aqueous solutions. As the potential becomes more negative, the concentration of Ca^2+^ and Cl^−^ near the working electrode will be higher and lower, respectively. When comparing the CaCl_2_·2H_2_O aqueous solution applied with a potential of −0.6 V to that applied with a potential of −0.2 V, the former one is more concentrated with respect to Ca^2+^ ions. Thus, the a_2_, b_2_ peak intensities of the former are lower than the latter. However, when no potential is applied, the Cl^−^ ion concentration is higher compared to any of the aqueous solutions applied with a negative potential. The presence of Cl^−^ ions at this interface results in a reduction in the number of solvent molecules in the first solvation shell surrounding the Ca^2+^ cations, similar to the concentrated (3 M) bulk solution, thus the a_2_, b_2_ peak intensities are lower than any of the aqueous solutions applied with a negative potential.

When positive potentials are applied, the overall spectra intensities become lower because of a significant reduction in the number of Ca^2+^ ions in the probing depth resulting from the electrostatic repulsions. Such electrostatic effect is stronger in aqueous solutions compared to that in methanol solutions. As it is shown in Fig. 5(b and c), when +0.2 V is applied, the overall peak intensity reduces to ∼1/4 of the peak intensity in the aqueous solution with no potential applied while the peak intensity decreases to ∼1/2 of the peak intensity in the methanol solution with no potential applied. This difference originates from the difference in the polarity and steric hinderance of the solvent molecules.

## Conclusions

In summary, a systematic study of the calcium solvation shell structure has been probed using L_3,2_-edge XAS, MD and DFT simulations. For CaCl_2_·2H_2_O aqueous solution, the intensity of the peaks is inversely correlated with the Ca^2+^ concentration because of the tetrahedral hydrogen-bonded network. In contrast, for that in methanol, the intensity remains similar while the concentration varies because only the first solvation shell is significant due to the weaker polarity and steric hinderance of CH_3_OH molecules compared to those of H_2_O. The effect of different potentials is also compared. The a_2_, b_2_ peak intensities are inversely correlated with the absolute value of the negative potentials applied on the working electrode while pre-edge a_1_, b_1_ peak intensities are similar. The effect of solvent is obvious when comparing 0 V and +0.2 V potentials applied for CaCl_2_·2H_2_O aqueous and methanol solutions. Such an understanding of calcium solvation at the atomic level will help depict its variation as a function of distance and electric field, contributing to the design of the next generation “beyond-lithium-ion” battery.

More importantly, this work involving the TEY mode L-edge soft XAS measured using the flow liquid cell enables to study the electrode/electrolyte interface while an electrochemical bias is applied. Although the coordination number obtained is similar to our previous study on the bulk of CaCl_2_ solution, in our other studies on Mg^2+^ and Zn^2+^ based electrolyte, we noticed that the interface and bulk can be different when an electrochemical bias is applied. Those results will be published in a separate study after a systematic investigation. Thus, it is of importance to characterize both the interface and the bulk properties to build an accurate and precise model of the liquid system, especially for battery and catalyst systems in which the reaction happens mainly at the interface. The use of the flow liquid cell allows electrochemical potentials to be applied, which shows implications in probing transient state species at the interface which can only be observed under *in situ*/operando conditions. The use of soft X-ray XAS to probe solvation structure of certain electrolytes can be applied in various different liquids. Here we chose CaCl_2_ in water and methanol as the model systems to investigate the difference between aqueous and non-aqueous solutions. The results indicate that the interface between liquid electrolyte and solid electrode can be different when electrochemical bias is applied. This is important for understanding the different behaviour of ions in aqueous/non-aqueous electrolytes for battery research.

As mentioned above, this setup using the flow liquid cell enables the *in situ*/operando probing in which the transient states might be observed. The TEY surface-sensitive signature makes it one of the very limited techniques that enable probing of such interfaces in future battery studies.

## Conflicts of interest

There are no conflicts to declare.

## Supplementary Material

RA-010-D0RA05905F-s001
