# Supplementary material for: Probing calcium solvation by XAS, MD and DFT calculations
Source: RSC Adv. 2020 Jul 21;10(46):27315–21. doi: 10.1039/d0ra05905f (PMC9055519; doi:10.1039/d0ra05905f)
Supplement: RA-010-D0RA05905F-s001 [file RA-010-D0RA05905F-s001.pdf]

Electronic Supplementary Material (ESI) for RSC Advances.
This journal is © The Royal Society of Chemistry 2020

Supporting Information

Probing calcium solvation by XAS, MD and DFT calculations

Feipeng Yang,[ab] Yi-Sheng Liu,[ab] Xuefei Feng,[ab] Kun Qian,[c] Li Cheng Kao,[b] Yang Ha,[b] Nathan T. Hahn,[ad] Trevor J Seguin,[ae] Mesfin Tsige,[c] Wanli Yang,[b] Kevin R. Zavadil,[ad] Kristin A. Persson,[aef] Jinghua Guo*[abg]

[a] Joint Center for Energy Storage Research, Lemont, IL 60439, USA.

[b] Advanced Light Source, Lawrence Berkeley National Laboratory, Berkeley, CA 94720, USA.

[c] Department of Polymer Science, The University of Akron, Akron, OH 44325, USA.

[d] Material, Physical and Chemical Sciences Center, Sandia National Laboratories, Albuquerque, NM 87185, USA.

[e] Energy Technologies Area, Lawrence Berkeley National Laboratory, Berkeley, CA 94720, USA.

[f] Department of Materials Science and Engineering, University of California Berkeley, Berkeley, CA 94720, USA.

[g] Department of Chemistry and Biochemistry, University of California Santa Cruz, Santa Cruz, CA 95064, USA.

Email: jguo@lbl.gov

MD simulation details:

The simulations were initially equilibrated using LAMMPS[1] under isothermal-isobaric (NPT) ensemble at a temperature of 300 K and a pressure of 1 atmosphere, followed by the canonical ensemble (NVT). A cut-off distance of 13 Å and 14 Å were used for nonbonded interactions in aqueous and methanol solution, respectively. For all electrostatic interactions, the mesh Ewald method was used[2]. Each of the simulations was run for at least 20 ns with a time step of 1fs and the last 1 ns data were used for analysis.

References:

1) S. Plimpton, *J. Comput. Phys.*, 1995, **117**, 1-19.

2) U. Essmann, L. Perera, M. L. Berkowitz, T. Darden, H. Lee and L. G. Pedersen, *J. Chem. Phys.*, 1995, **103**, 8577–8593.

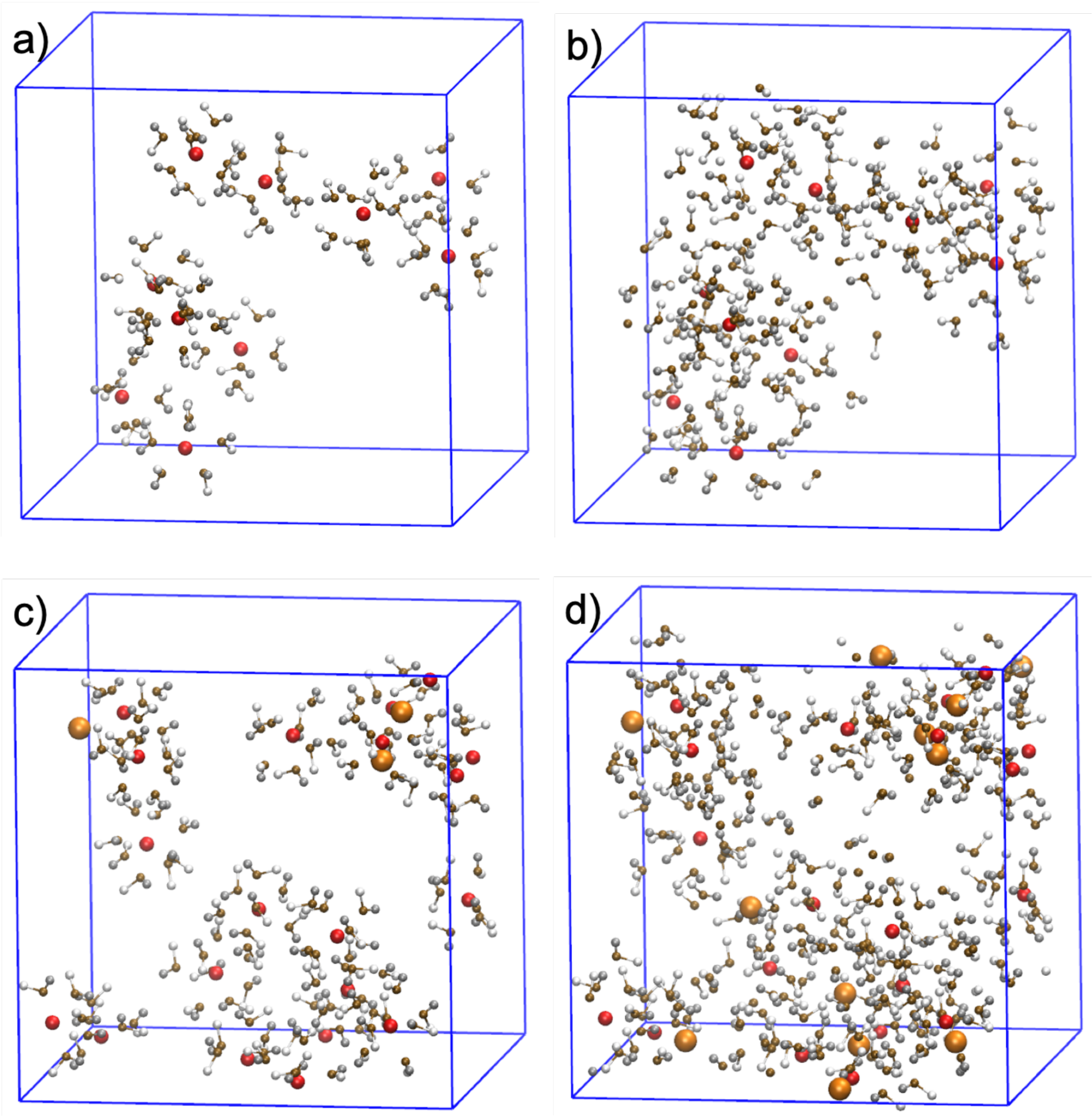

Figure S1 Molecular dynamics (MD) simualtion for (a) the first solvation shell around Ca^{2+} in 0.5 M $CaCl_2{\cdot}2H_2O$ methanol solution; (b) the first and second solvation shells around Ca^{2+} in 0.5 M $CaCl_2{\cdot}2H_2O$ methanol solution; (c) the first solvation shell around Ca^{2+} in 1.0 M $CaCl_2{\cdot}2H_2O$ methanol solution; (d) the first and second solvation shells around Ca^{2+} in 1.0 M $CaCl_2{\cdot}2H_2O$ methanol solution. (Red: Ca^{2+}, Orange: Cl^-)

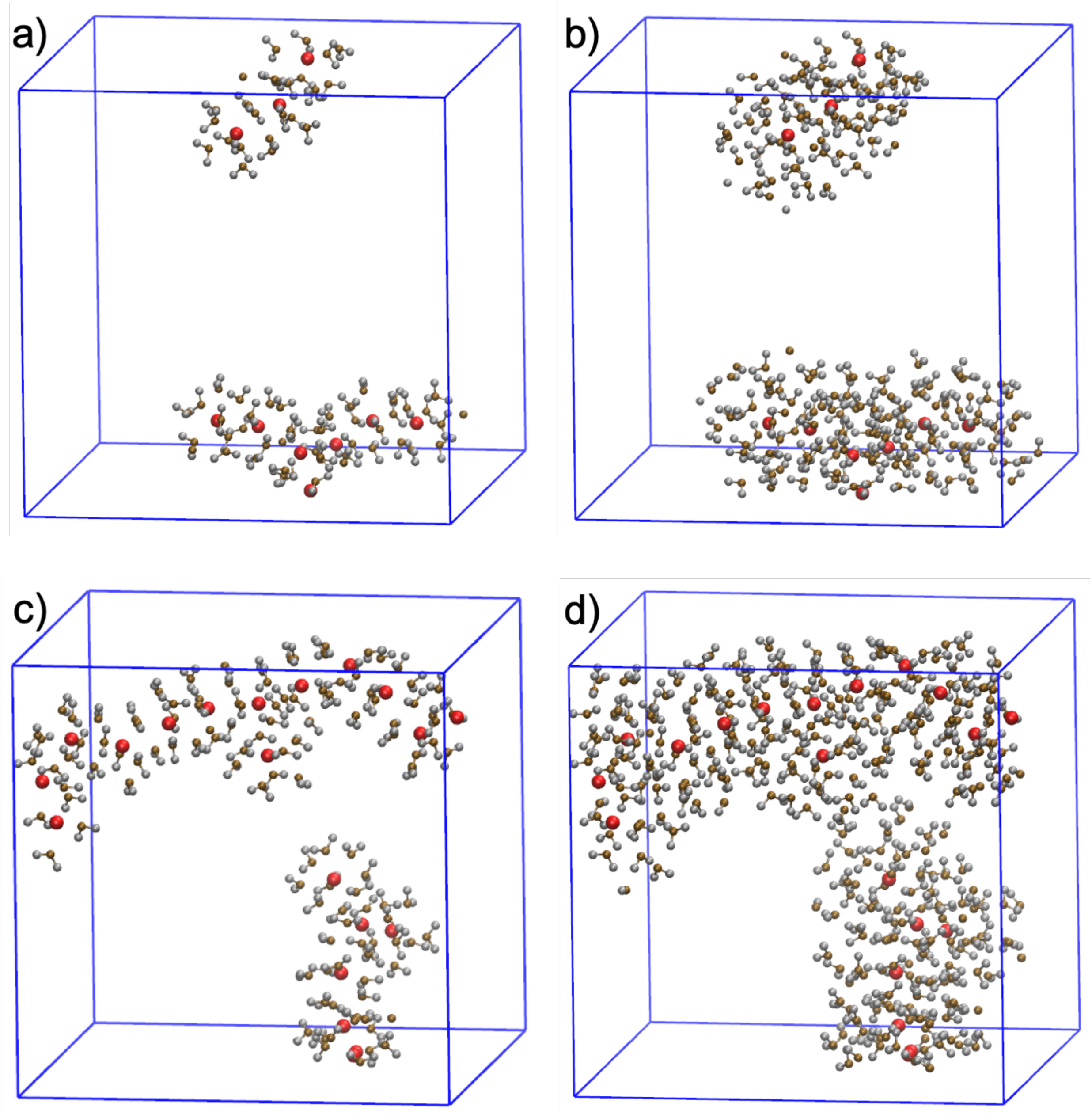

Figure S2 Molecular dynamics (MD) simualtion for (a) the first solvation shell around Ca^{2+} in 0.5 M $CaCl_2 \cdot 2H_2O$ aqueous solution; (b) the first and second solvation shells around Ca^{2+} in 0.5 M $CaCl_2 \cdot 2H_2O$ aqueous solution; (c) the first solvation shell around Ca^{2+} in 1.0 M $CaCl_2 \cdot 2H_2O$ aqueous solution; (d) the first and second solvation shells around Ca^{2+} in 1.0 M $CaCl_2 \cdot 2H_2O$ aqueous solution.

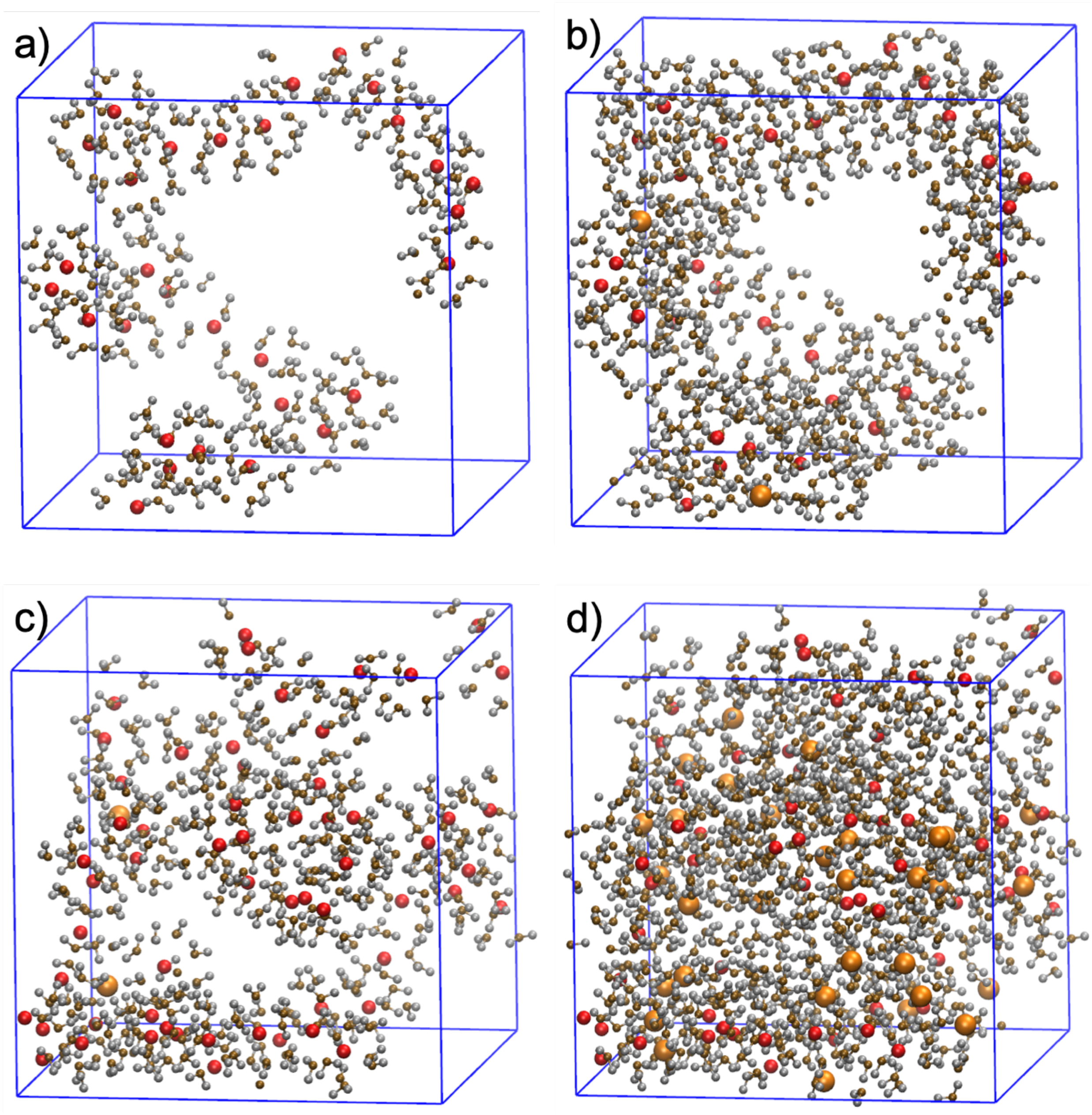

Figure S3 Molecular dynamics (MD) simualtion for (a) the first solvation shell around Ca^{2+} in 1.5 M $CaCl_2 \cdot 2H_2O$ aqueous solution; (b) the first and second solvation shells around Ca^{2+} in 1.5 M $CaCl_2 \cdot 2H_2O$ aqueous solution; (c) the first solvation shell around Ca^{2+} in 3.0 M $CaCl_2 \cdot 2H_2O$ aqueous solution; (d) the first and second solvation shells around Ca^{2+} in 3.0 M $CaCl_2 \cdot 2H_2O$ aqueous solution.
